# Gender differences in asthma perception and its impact on quality of life: a post hoc analysis of the PROXIMA (Patient Reported Outcomes and Xolair^®^ In the Management of Asthma) study

**DOI:** 10.1186/s13223-019-0380-z

**Published:** 2019-11-06

**Authors:** Delia Colombo, Emanuela Zagni, Fabio Ferri, Giorgio Walter Canonica, the PROXIMA study centers, Corrado Astarita, Piero Balbo, Marialma Berlendis, Giacomo Bruni, Caterina Bucca, Giorgio Walter Canonica, Angelo Guido Corsico, Antonio Foresi, Bruno Macciocchi, Giovanni Michetti, Mariacarmela Montera, Paolo Palange, Carlo Pareo, Biagio Polla, Riccardo Polosa, Enrico Puddu, Paola Rogliani, Antonino Romano, Paola Rottoli, Eugenio Sabato, Pierachille Santus, Domenico Schiavino, Antonio Spanevello, Angelo Trovato, Maria Cristina Zappa

**Affiliations:** 1 Novartis Farma S.p.A, Largo Umberto Boccioni, 1, 21040 Origgio, Varese Italy; 2 Medineos Observational Research, Modena, Italy; 3 Department of Biomedical Sciences, Personalised Medicine Clinic Asthma & Allergy, Humanitas University, IRCCS Humanitas Research Hospital, Rozzano, Milan, Italy

**Keywords:** Omalizumab, Severe allergic asthma, Asthma control, Quality of life, Disease perception, Gender differences

## Abstract

**Background:**

Gender differences in asthma perception and control have been reported. The PROXIMA observational study assessed these outcomes in a cohort of Italian severe allergic asthma (SAA) patients. This post hoc analysis of the PROXIMA results was aimed at assessing gender differences in SAA in a real-world setting, focusing on disease perception and impact on quality of life (QoL).

**Methods:**

The PROXIMA study was an observational, multicenter study, consisting of a cross-sectional and a prospective longitudinal phase, including adult outpatients diagnosed with SAA at step 4 requiring a therapeutic step-up. Patients on omalizumab treatment at baseline were included in the 12-month longitudinal phase. Disease control was assessed by the Asthma Control Questionnaire (ACQ) score, patients’ disease perception by the Brief Illness Perception Questionnaire (BIPQ), and QoL by the EuroQoL five-dimensional three-level questionnaire (EQ-5D-3 L) at baseline and after 6 and 12 months. Two regression models were used to evaluate the association between gender and BIPQ total score and EQ-5D-3L score, respectively.

**Results:**

357 patients (65% females) were analyzed for the cross-sectional phase and 99 (62.6% females) for the longitudinal phase. The prevalence of perennial and seasonal aeroallergens was similar between genders. ACQ score decreased similarly during omalizumab treatment at 6 and 12 months in both genders; no gender differences were observed in control rates. Asthma perception was worse among females at all study visits reaching statistical significance at 12 months (mean (SD) B-IPQ total score 41.8 (9.4) vs 35.6 (12.0); *T* test p-value (males vs females) < 0.05). Statistically significant gender differences were observed for some specific items, with males reporting less symptom experience, concern about the disease, and emotional impact at 12-months. The results of the multivariate regression model for repeated measures showed that overall treatment with omalizumab improved disease perception overtime regardless from gender. Males reported a significantly better QoL compared to females at both 6 and 12 months.

**Conclusions:**

In this real-world setting, females confirmed to have a worse perception of asthma, feel it as more symptomatic and suffer a greater impact on their QoL, even though having similar baseline severity and obtaining similar level of control.

## Background

Asthma is a chronic disease that affects more than 300 million people of all ages and ethnic groups worldwide and represents a major health care burden [1]. Furthermore, its prevalence is increasing together with that of allergic diseases: in Italy, in particular, the asthma epidemic does not seem to slow down, with an increase in prevalence of 38% reported between 1990 and 2010, in parallel with a similar increase in asthma-like symptoms and allergic rhinitis [2]. Severe asthma affects 5–10% of all asthma patients [3], often remaining uncontrolled despite high-dose inhaled corticosteroids (ICS) and long-acting beta_2_-agonists (LABA). Among adults, the incidence and severity of asthma are higher in females than in males, particularly between the 4th and 6th decade [4, 5]; over the age of 35, asthma was reported to be 20% more frequent in females than in males [6]. On the other hand, before puberty boys are more frequently affected than girls [4, 7] and in the elderly the differences in prevalence tend to decrease [8]. Although the basis for these gender differences are not yet fully understood, several hypotheses have been made. The above reported trends in asthma prevalence suggest that sex hormones are implicated in asthma pathogenesis, with female sex hormones and their receptors favoring asthma development and male sex hormones and their receptors exerting a protective effect. Some authors have suggested a greater hyper-responsiveness in females than in males [9], others have underlined differences between genders in lung capacity [10, 11]. Differences have also been reported in perception of air flow obstruction, with females complaining significantly lower general health status, more symptoms and more activity limitations [12, 13], and in health-related quality of life (QoL) [14, 15].

The PROXIMA (Patient Reported Outcomes and Xolair^®^ In the Management of Asthma) study was an observational, non-controlled, multicenter, two-phase study in Italian severe allergic asthma (SAA) patients, aimed at assessing the prevalence of perennial environmental allergies (during the cross-sectional phase) and the rate of disease control after up to 12 months of omalizumab treatment (during the longitudinal phase). The secondary objectives of the study included the assessment of patients’ disease perception and level of asthma control during both the cross-sectional and longitudinal phases and patients’ compliance to and persistence with omalizumab, rate of exacerbations and patients’ QoL during the longitudinal phase [16, 17].

Novartis has put in place a wide gender-medicine project, called MetaGeM, with the purpose to evaluate gender differences in clinical outcomes, therapeutic approaches and safety parameters, by means of post hoc analyses and meta-analyses of previously conducted observational trials. The MetaGeM project includes 11 Italian observational studies in different clinical areas, including immune-mediate disorders, organ transplants, and infectious, central nervous system, cardiovascular and respiratory diseases, performed between 2002 and 2016. Within the MetaGeM project, a post hoc analysis of the results of both phases of the PROXIMA study has been conducted with the aim of exploring gender differences in SAA in a real-world setting, with particular focus on disease perception and impact on patients’ QoL. We here report the results of this post hoc gender analysis aimed to evaluate in the PROXIMA SAA population (i) the association between gender and presence of perennial or seasonal allergies at baseline and, in patients on omalizumab treatment included in the longitudinal phase, the effect of gender on (ii) asthma control, (iii) asthma perception and (iv) QoL during 12 months of follow up.

## Methods

The PROXIMA study was an observational, non-controlled, multicenter cohort study, conducted from December 27, 2013 to June 21, 2016 at 25 Italian centers specialized in asthma treatment, consisting of two phases, a cross-sectional and a prospective longitudinal phase. Outpatients aged ≥ 18 years, diagnosed with SAA at step 4 according to GINA 2012 guidelines and requiring a therapeutic step-up [18, 19], were included in the cross-sectional phase. Patient unable to complete the patient questionnaires or included in an experimental study at entry were excluded.

Those who were on treatment with omalizumab at baseline visit were included in the 12-month longitudinal phase, provided that they had started omalizumab as per clinician judgement (according to AIFA criteria) not earlier than 15 days before enrolment and within 30 days after enrolment.

In the PROXIMA study, the level of disease control had been assessed by the Asthma Control Questionnaire (ACQ) score, a validated tool with a 7-point scale, where 0 means well-controlled and 6 extremely poorly controlled asthma [20]. According to Van den Nieuwenhof L et al. [21], an ACQ total score between 0 and 4 indicates a good to moderate symptom control and a score ≥ 4 a poor/very poor symptom control. Patients with an ACQ score < 4 at either 6 or 12 months were so classified as “controlled” and patients with a score of ≥ 4 as uncontrolled.

Patient’s disease perception had been assessed by means of the Brief Illness Perception Questionnaire (B-IPQ), a validated 9-item questionnaire designed to rapidly assess cognitive and emotional representations of illness [22]. Five of the items assess cognitive illness representations (consequences, Item 1; timeline, Item 2; personal control, Item 3; treatment control, Item 4; and identity, Item 5), two assess emotional representations (concern, Item 6 and emotions, Item 8) and one assesses the level of illness comprehension (Item 7). Assessment of the causal representation (Item 9) is by an open response item, which asks patients to list what they consider the three most important causal factors in their illness. All the questionnaire items (except item 9) are rated using a 1-to-10 response scale; for all items 1 means best and 10 worst perception of the disease with the exception of items 3, 4 and 7 where the score is reverse (i.e. 1 means worst and 10 best perception of the disease). B-IPQ total score ranges between 8 and 80, with a higher score reflecting a worse perception of the disease; it was calculated by summing the responses to items 1 to 8 (after reversing the score for items 3, 4 and 7).

The patients’ QoL had been measured by the EuroQoL five-dimensional three-level questionnaire (EQ-5D-3L), a validated and standardized measure of health status developed by EuroQol Group to provide a simple generic measure of health status for clinical and economic evaluation [23, 24]. All these evaluations had been performed at baseline and after 6 and 12 months. Detailed study design, objectives, and overall results have been reported elsewhere [16, 17].

### Statistical methods

Being this a post hoc analysis, all patients evaluated in the cross-sectional and longitudinal phases of the PROXIMA study were considered in the gender analysis, with no formal a priori hypothesis about gender distribution of patients. For the cross-sectional phase, all patients fulfilling inclusion/exclusion criteria were analyzed; for the longitudinal phase patients treated with at least one dose of omalizumab at study entry (from 15 days before to 30 days after enrolment) who completed the longitudinal phase on treatment with omalizumab (i.e. who did not discontinue omalizumab treatment during the whole follow-up period) and with available ACQ assessment at baseline and during the follow-up period were considered.

The continuous, normally distributed variables were expressed as a mean ± SD and comparisons between groups were performed with the parametric Student’s t test. In case of not normally distributed parameters, median and interquartile range (IQR) were provided and males were compared to females by means of the non-parametric Wilcoxon rank sum test. Absolute and relative frequencies were calculated for qualitative data and differences between categorical variables were tested by Chi square or Fisher exact test. The significance threshold was set as 0.05 (all p-values presented are exploratory, so no correction for multiple testing was applied [25]). Missing values were not replaced and did not contribute to the analysis of the variable.

Eight bivariate logistic regression models were used to assess the association between perennial asthma and gender, keeping constant the effect of the following clinically relevant covariates: age, duration of asthma, age at diagnosis and serum IgE level (included in the model as continuous variable), presence of comorbidities (yes; no), number of exacerbations during the last year (0; ≥ 1), smoking (current/former smoker; no smoker), and ACQ score (< 4; ≥ 4).

Two regression models for repeated measures—since outcomes were assessed at baseline and follow-up visits—were used to evaluate the effect of gender on (1st model) patients’ disease perception (B-IPQ total score) and (2nd model) QoL (EuroQoL score), keeping constant the effect of other clinically relevant covariates. As covariates the following were included: age and duration of asthma (as continuous variable), presence of comorbidities at baseline visit (yes; no), smoking at baseline (current/former smoker; no smoker), number of exacerbations in the year before baseline (0; ≥ 1), total omalizumab dose taken during follow-up (as continuous variable), concomitant medications with ICS, ICS + LABA, oral corticosteroids (OCS) (yes; no). We were also interested in estimating the effect of time on patients’ disease perception and QoL, holding gender and all the other variables included in the model constant. For this reason, the visit (baseline, 6-month follow up, 12-month follow-up) was also included as covariate in the two models.

Statistical analysis was performed using SAS v9.4 and Enterprise Guide v7.1. Project management including data banking, quality control and statistical analysis, was performed by Medineos Observational Research (Modena, Italy).

## Results

The analyzed population included 357 patients in the cross-sectional phase—232 (65.0%) females—and 99 patients in the longitudinal phase—62 (62.6%) females. Median (IQR) age at study inclusion was 52.3 (40.2–61.9) years in females and 50.2 (38.1–64.5) years in males. Demographic and baseline clinical characteristics by gender and study phase are summarized in Table 1. The only significant difference was registered in FEV1, which was lower in females than in males. The prevalence of perennial and seasonal aeroallergens in the cross-sectional population was 87.4% vs 12.6% respectively in females and 92.6% vs 7.4% respectively in males (Chi square test, p = 0.1415), showing no association between gender and presence of perennial or seasonal allergy. Bivariate logistic regression models showed no relevant association between perennial asthma and gender too (Table 2); the association between gender and perennial asthma keeping constant the presence of exacerbations during the year before baseline (OR of perennial asthma females vs males 0.362, 95% CI 0.143–0.918), in fact, could be influenced by data distribution.

**Table 1:** Baseline demographic and clinical characteristics by gender

	Cross-sectional population (N = 357)				p-value*	Longitudinal population (N = 99)				p-value*
	Females (N = 232, 65.0%)		Males (N = 125, 35.0%)			Females (N = 62, 62.6%)		Males (N = 37, 37.4%)		
Age (years)	52.3 (40.2–61.9)		50.2 (38.1–64.5)		0.648	52.3 (11.9)		51.0 (15.8)		0.640
Smoking habits (n (%))										
Current smoker	17 (7.3)		8 (6.4)		0.430	3 (4.8)		2 (5.4)		0.795
Former smoker	39 (16.8)		28 (22.4)			10 (16.1)		8 (21.6)		
Menopausal status (n (%))	114 (49.1)					32 (51.6)				
Comorbidities (n (%))										
≥ 1	138 (59.5)		70 (56.0)		0.524	38 (61.3)		27 (73.0)		0.236
Cardiovascular disease	48 (20.7)		26 (20.8)		0.980	13 (21.0)		5 (13.5)		0.352
Chronic rhinitis	35 (15.1)		17 (13.6)		0.704	11 (17.7)		5 (13.5)		0.580
Chronic sinusitis/rhinosinusitis	22 (9.5)		15 (12.0)		0.457	11 (17.7)		8 (21.6)		0.635
GERD	38 (16.4)		13 (10.4)		0.124	8 (12.9)		6 (16.2)		0.647
Hormonal disturbances	27 (11.6)		16 (12.8)		0.748	8 (12.9)		4 (10.8)		1.000
Nasal polyps	16 (6.9)		11 (8.8)		0.516	8 (12.9)		7 (18.9)		0.419
Obesity	19 (8.2)		4 (3.2)		0.067	5 (8.1)		0 (0.0)		0.154
Psychiatric disorders^a^	14 (6.0)		3 (2.4)		0.124	4 (6.5)		1 (2.7)		0.648
Asthma history										
Duration (years)	14.8 (5.7–26.8)	n = 226	15.2 (5.3–29.8)	n = 121	0.849	16.6 (8.1–29.2)	n = 60	14.6 (5.0–26.3)	n = 36	0.280
Age at diagnosis (years)	33.6 (20.9–44.5)	n = 226	31.1 (14.7–44.6)	n = 121	0.199	32.1 (15.7)	n = 60	34.1 (15.6)	n = 36	0.555
No of asthma exacerbations in the last 12 months	2.0 (1.0–4.0)	n = 213	2.0 (1.0–4.0)	n = 117	0.366	4.0 (2.0–6.0)	n = 60	4.0 (2.0–6.0)	n = 35	0.425
≥ 1 exacerbation in the 12 months before baseline (n (%))	191 (89.7)	n = 213	98 (83.8)	n = 117	0.119	58 (96.7)	n = 60	34 (97.1)	n = 35	1.000
FEV1 (L)	1.8 (1.2–2.2)	n = 223	2.3 (1.7–3.0)	n = 119	*<* *0.0001*	1.4 (1.1–1.8)	n = 61	2.1 (1.4–2.6)	n = 36	*0.001*
Serum IgE (IU/mL)	216.0 (101.0–525.0)	n = 164	294.0 (150.5–629.0)	n = 88	0.054	238.0 (123.8–499.0)	n = 60	294.0 (168.0–540.0)	n = 37	0.399

Mean (SD) were showed for continuous, normally distributed variables and comparisons between groups were performed with parametric Student’s t test. In case of not normally distributed parameters, median (IQR) were provided and males were compared to females by means of non- parametric Wilcoxon rank sum test

If not otherwise specified, number of analyzed patients is reported in table heading

*GERD* Gastroesophageal reflux

^a^Anxiety, depression, behavioral disorders

*Student’s t test or Wilcoxon rank sum test p-values were showed for numerical variables; Chi square or Fisher exact test p-values were showed for categorical ones. Statistically significant p-values are in italic

**Table 2:** Association between gender and perennial asthma: bivariate logistic regression models adjusted for relevant covariates

		OR gender (Females vs males) [95% CI]	OR other covariates* [95% CI]
*Age at baseline (years)	n = 335	0.556 [0.252–1.227]	0.988 [0.965–1.011]
*Presence of comorbidity at baseline (no vs yes)	n = 335	0.550 [0.249–1.213]	0.705 [0.352–1.414]
*Asthma duration at baseline (years)	n = 325	0.541 [0.244–1.199]	0.986 [0.965–1.009]
*Age at diagnosis (years)	n = 325	0.558 [0.253–1.232]	1.001 [0.981–1.021]
*N° exacerbations (0 vs ≥ 1)	n = 308	0.362 [0.143–0.918]	0.470 [0.176–1.258]
*IgE serum level at baseline (IU/mL)	n = 238	0.301 [0.085–1.063]	1.002 [1.000–1.003]
*Smoke (Current/former vs no smoker)	n = 335	0.564 [0.256–1.244]	1.425 [0.599–3.394]
*ACQ score (< 4 vs ≥ 4)	n = 318	0.534 [0.233–1.227]	0.845 [0.243–2.947]

The model estimates the odds ratio of having perennial asthma

95% Wald Confidence Limits are showed

In the original PROXIMA study the mean (SD) compliance rate to omalizumab (assessed in terms of proportion of administered over the total number of planned injections) was 96.9 (7.8)% during the 12-month follow-up. In the longitudinal phase, the mean (SD) total dose of omalizumab—whose posology depends on serum IgE level and body weight—injected during the follow-up period was 6309.3 (3820.1) mg in females and 6934.5 (4148.4) mg in males. Asthma medications taken concomitantly to omalizumab are summarized in Table 3. Females were taking overall more OCS in different combinations (18.5% in females vs 8.8% in males) and less inhaled therapies—ICS alone or plus ICS/LABA fixed combination without OCS—than males (79.6% in females vs 91.2% in males). However, no statistically significant differences by gender emerged.

**Table 3:** Ongoing asthma therapies concomitant to omalizumab during the observational period by gender

Treatment regimen	Females (N = 54) n (%)	Males (N = 34) n (%)	p-value*
No ongoing therapies	1 (1.9)	0 (0.0)	0.281
ICS	43 (79.6)	31 (91.2)	
ICS only	3 (5.6)	3 (8.8)	
ICS/LABA only	33 (61.1)	21 (61.8)	
ICS + ICS/LABA	7 (13.0)	7 (20.6)	
OCS	10 (18.5)	3 (8.8)	
ICS/LABA + OCS	5 (9.3)	1 (2.9)	
ICS + ICS/LABA + OCS	3 (5.6)	0 (0.0)	
ICS + OCS	2 (3.7)	2 (5.9)	

Only therapies that were ongoing at baseline and at 6 and 12 months were considered.ICS: inhaled corticosteroids; ICS/LABA: fixed combination of ICS and LABA; LABA: long acting beta_2_-agonists; OCS: oral corticosteroids

*P-value of Fisher exact test treatment regimen (no ongoing therapies, ICS, OCS) vs gender (females, males) is showed

**Fig. 1:**
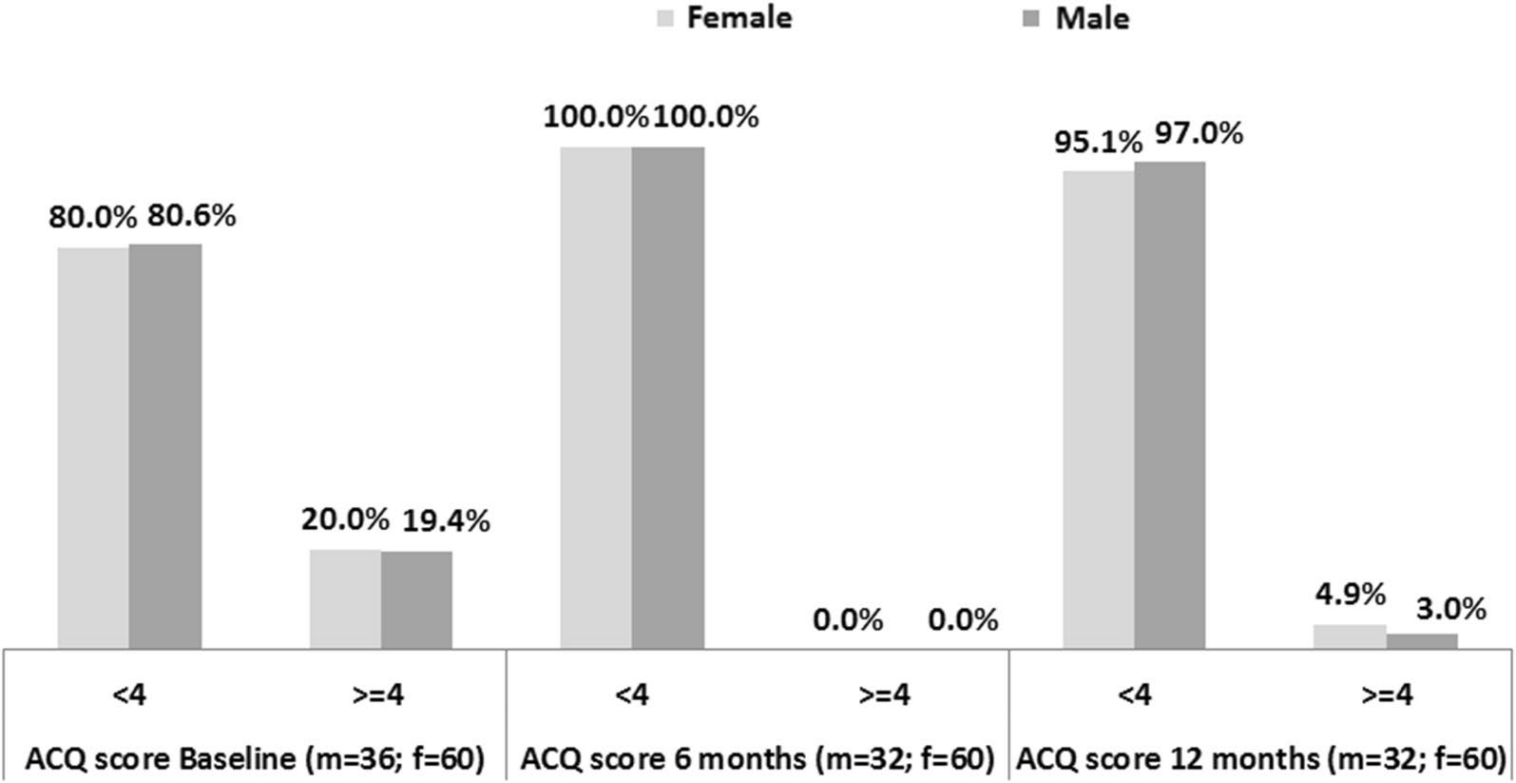
Patients with controlled (ACQ < 4) and uncontrolled (ACQ ≥ 4) asthma by gender. Chi square/Fisher test (% of controlled patients vs gender) p-value > 0.05. The test was not performed at 6 months because all patients had controlled asthma

Median ACQ score, expressing the level of asthma control (the lower the score, the greater the control), decreased from baseline both at 6 and 12 months in males and females (Table 4). Considering the score of 4 as the cut-off for controlled asthma [20], as shown in Fig. 1, no gender differences were observed in the control rates, both at baseline and during follow-up (Chi square/Fisher test % of controlled patients vs gender p-value > 0.05; test not performed at 6 months because all patients had controlled asthma).

**Table 4:** Asthma Control Questionnaire (ACQ) score by gender, at baseline and after 6 and 12 months

	N	Median (IQR)
Baseline		
Female	60	3.0 (2.1–3.6)
Male	36	2.9 (1.9–3.7)
6 months		
Female	60	1.3 (0.6–2.4)
Male	32	1.1 (0.6–1.5)
12 months		
Female	61	1.4 (1.0–2.4)
Male	33	1.1 (0.4–1.7)

The B-IPQ scores at all time points by gender for all items and total score are reported in Table 5. Asthma perception was slightly worse among females than males at all study visits reaching statistical significance at 12-month follow-up visit (mean (SD) B-IPQ total score 41.8 (9.4) in females vs 35.6 (12.0) in males; T-test p-value (males vs females) < 0.05). Statistically significant gender differences were observed for some specific items, with males reporting less symptom experience (item 5), less concern about the disease (item 6), and less emotional impact (item 8) than females at 12-month follow-up. Moreover, males felt to better understand their illness at baseline (item 7) (see Table 5). The results of the multivariate regression model for repeated measures showed that overall treatment with omalizumab improved disease perception overtime regardless from gender. In fact, no effect of gender on B-IPQ total score exists (beta estimate females vs males: 2.83, p-value beta estimate > 0.05) and the B-IPQ total score was approximately 10 points lower at 6 and 12 months of follow-up compared to baseline (beta estimate 6-month follow up visit vs baseline visit and beta estimate 12-month follow up visit vs baseline visit: − 10.97, p-value beta estimate < 0.0001).

**Table 5:** Mean B-IPQ scores by gender at baseline and at 6 and 12 months

Items	Baseline			6 months			12 months		
	n		p-value*	n		p-value*	n		p-value*
1. Consequences: How much does your illness affect your life?									
Females	61	7.3 (1.9)	0.830	61	5.0 (2.4)	0.304	62	5.1 (2.6)	0.292
Males	37	7.2 (1.9)		35	5.6 (2.9)		36	4.6 (2.8)	
2. Timeline: How long do you think your illness will continue?									
Females	60	8.3 (2.2)	0.229	59	7.4 (2.5)	0.466	60	8.0 (2.5)	0.446
Males	37	7.7 (2.5)		35	7.0 (3.2)		36	7.6 (2.7)	
3. Personal control: How much control do you feel you have over your illness?									
Females	61	5.9 (1.9)	0.354	61	6.4 (2.0)	0.177	62	6.8 (2.0)	0.351
Males	37	6.3 (2.0)		35	7.0 (1.9)		36	7.2 (2.5)	
4. Treatment control: How much do you think your treatment can help your illness?									
Females	61	7.0 (2.2)	0.616	61	7.8 (2.3)	0.098	62	*8.0 (2.0)*	*0.031*
Males	37	7.2 (2.6)		35	8.5 (1.5)		36	*8.7 (1.4)*	
5. Identity: How much do you experience symptoms from your illness?									
Females	61	7.6 (2.0)	0.964	60	5.4 (2.6)	0.503	62	*5.9 (2.4)*	*0.026*
Males	37	7.5 (1.5)		35	5.0 (2.7)		36	*4.8 (2.5)*	
6. Concern: How concerned are you about your illness?									
Females	61	7.2 (2.5)	0.490	61	5.4 (2.7)	0.519	62	*6.1 (2.8)*	*0.039*
Males	36	7.6 (2.3)		35	5.8 (2.7)		36	*4.9 (2.7)*	
7. Illness comprehension: How well do you feel you understand your illness?									
Females	61	*6.7 (2.4)*	*0.038*	60	7.3 (2.1)	0.118	62	7.6 (2.1)	0.790
Males	37	*7.7 (2.1)*		35	8.0 (2.2)		36	7.8 (2.3)	
8. Emotions: How much does your illness affect you emotionally?									
Females	61	7.0 (2.8)	0.305	60	5.7 (2.8)	0.492	62	*6.2 (2.7)*	*0.005*
Males	37	6.5 (2.6)		35	5.3 (2.5)		36	*4.6 (2.6)*	
Total score									
Females	60	50.9 (8.1)	0.154	58	40.6 (9.8)	0.287	60	*41.8 (9.4)*	*0.007*
Males	36	48.4 (8.3)		35	38.2 (11.4)		36	*35.6 (12.0)*	

Mean (SD) were showed

B-IPQ items range between 1 and 10; for all items 1 means best and 10 worst perception of the disease with the exception of items 3, 4 and 7 where score is reversed (and so 1 means worst and 10 best perception of the disease)

B-IPQ total score ranges between 8 and 80, with a higher score reflecting a worse perception of the disease

*Student’s t test p-values were showed. Statistically significant p-values are in italic

**Fig. 2:**
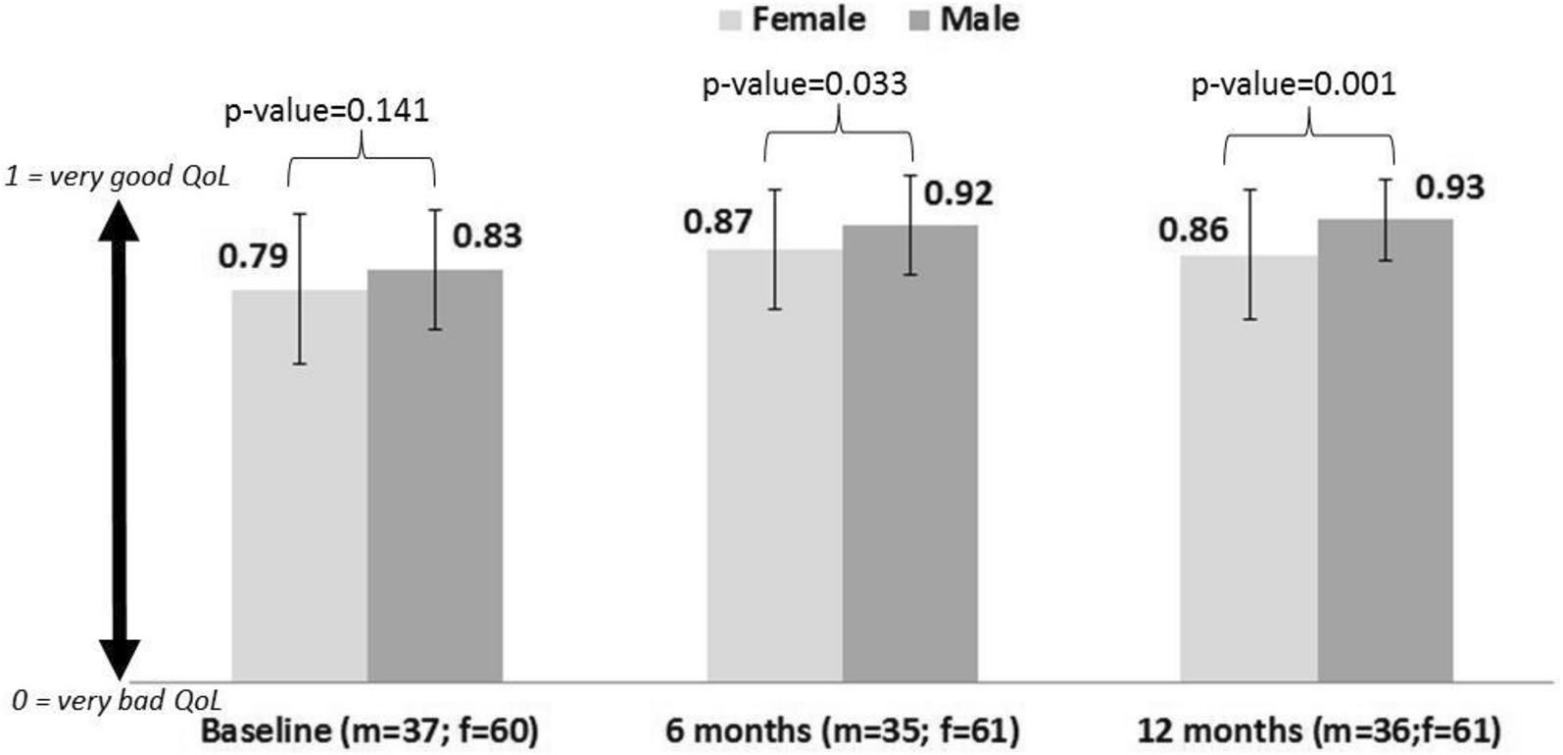
Mean EuroQoL score by gender at baseline and at 6 and 12 months of follow-up. Mean and SD of EuroQol score are showed. *T-test (EuroQol score males vs females) p < 0.05

Gender differences were observed in EuroQoL score at study visits, with males reporting a better QoL that reached statistical significance in comparison to females at 6 and 12 months (Fig. 2). This was confirmed by the results of the model for repeated measures (Table 6), showing that gender and time significantly impact on the EuroQoL score; in fact, females had a score 0.055 lower than males and the score improves 0.1 point from baseline to 12-month follow up, irrespectively of gender.

**Table 6:** Repeated measures multivariate regression: effect of gender on EuroQol score

	Estimate	Standard Error	p-value
Intercept	0.820	0.071	< 0.0001
Gender (females vs males)	− 0.055	0.024	0.028
Visit (6-month follow up vs baseline)	0.093	0.015	< .0.0001
Visit (12-month follow up vs baseline)	0.099	0.015	< .0.0001
Age (years)	0.000	0.001	0.826
Comorbidities (no vs yes)	0.037	0.025	0.147
Smoke (current/former vs no smoker)	− 0.009	0.028	0.752
Asthma duration (years)	0.000	0.001	0.712
ICS (no vs yes)	− 0.026	0.029	0.382
ICS + LABA (no vs yes)	− 0.032	0.041	0.439
OCS (no vs yes)	0.039	0.036	0.278
Omalizumab total dose (mg)	0.000	0.003	0.510
Exacerbations in the 12 months before enrollment (0 vs ≥ 1)	0.052	0.063	0.409

## Discussion

This post hoc analysis of the PROXIMA study evaluated gender differences in severe asthma patients in a real-world setting, assessing control rates following add-on therapy with omalizumab and focusing on disease perception and asthma impact on QoL. The PROXIMA study found that among SAA patients, perennial allergies were highly prevalent (95.8% based on clinical judgment; 83.8% based on confirmatory allergy test). In our by-gender analysis, no significant difference was found in the prevalence of perennial vs seasonal allergies between males and females. Our results show that in the PROXIMA patient population, defined by the requirement of step-up therapy at enrolment, males and females obtained similar level and rates of asthma control with add-on omalizumab, as measured by the ACQ. However, the overall perception of the disease, assessed by the B-IPQ, though improving on omalizumab treatment, was significantly worse in females compared to males at 12-month follow-up visit. In particular, some aspects of the disease were perceived significantly worse in females, i.e. symptom experience, concern about asthma, and emotional impact. We were not able to find in the literature whether these differences in the B-IPQ score translates into a clinically relevant difference and this can be considered a limitation of the B-IPQ score. However, our results are consistent with previous observations in patient populations of different countries, reporting that females tend to complain more bothersome day- and night-time asthma symptoms than males and have a worse perception of such symptoms and of disease control, despite having similar pulmonary function and asthma medications [15, 26, 27]. Lee and colleagues observed that females were more likely to report nocturnal awakenings and missed activities, also when having better pulmonary function than males [28]. It is not uncommon, especially in asthma, that clinical improvement may not correlate with patient’s experience [29] and this may explain the different disease perception observed in males and females having obtained similar levels of disease control. Males reported the feeling of a better understanding of their asthma from study beginning, showing to be more aware of their condition. Consistently with the perception results, in our study gender showed to exert a significant effect on patients’ QoL, measured by EQ-5D-3 L. While QoL improved in both gender after 6 and 12 months of omalizumab add-on compared to baseline, significant gender differences emerged at both follow-up visits, with females reporting a lower QoL than males. According to the review by Coretti et al. [30], the minimal clinically important difference (MCID) for the EQ-5D index ranges from 0.03 to 0.54; therefore, the difference registered in our study between males and females can be considered somewhat clinically relevant. Thus, also in terms of asthma impact on QoL, females showed to be more heavily affected by the disease. In this respect, our results are in line with several previous studies demonstrating that females frequently report worse asthma related QoL [14, 15, 27, 31, 32]. However, it should be underlined that asthma was more severe in females from baseline, as measured by the FEV1, which was significantly lower in females.

One peculiar aspect of the PROXIMA study was precisely the evaluation, as secondary objectives, of the perceived burden of disease by means of patient reported outcomes (PROs) [17]. Indeed, the patient’s point of view is increasingly becoming an important part of treatment evaluation. However, only a minority of asthma trials have included PROs among their assessment measures and even less in a real-life setting [33]. In this by-gender analysis of the PROXIMA real-life results, PROs are exactly what makes the difference between males and females with severe asthma. Patients of both genders achieved similar levels of asthma control and experienced overall improvement in QoL over 1 year of omalizumab add-on therapy, but females reported a worse overall perception of the disease and complained worse symptom experience and greater emotional impact. Moreover, they expressed more concern about the disease and felt a greater impairment of their QoL, impacting their usual daily activities and producing more discomfort and anxiety/depression, as explored by EQ-5D-3 L. Female sex hormones are hypothesized to affect these outcomes [10, 34], however other factors have been claimed, such as different behaviors of asthmatic males and females [34]—females have shown a lower threshold for healthcare contact requirement [35]—, different adherence to medications—females seem to need more encouragement and education that males regarding the correct use of inhalers [36]—, and different attitudes of caregivers toward males and females—with for example females undergoing less spirometry testing [37]. Concerning medications, it may be interesting to report that our female population showed a trend toward a greater use of OCS and a lower use of pure inhaled medications.

This study has the important limitation of being a post hoc analysis of a study that had not originally been designed with the aim of detecting gender differences. It also carries the limitation of the original PROXIMA study of a higher numerical imbalance between sexes than reported in the literature. Furthermore, the use of questionnaire in the PROXIMA study may have implied a potential risk of selection bias owing to exclusion of patients who were considered unable to complete them. Another limitation is that, despite the choice of standardized questionnaires, several PROXIMA findings were based on self-report and thus subject to recall bias. On the other hand, our post hoc gender analysis has the merit of providing real-world information about the differences in PROs from males and females with severe asthma. The absence of spirometry data at the 6 and 12 months, that would have allowed to correlate objective measures of respiratory function of our SAA patients to their ACQ and QoL scores, is another limitation due to the post hoc nature of our study.

## Conclusions

In this real-world setting, females confirmed to have a worse perception of the disease, feel the disease as more symptomatic and suffer a greater impact on their daily activities and QoL, even though having similar baseline severity and obtaining similar level of disease control. As emerged from other MetaGeM post hoc analyses, the impact of conditions and therapies on males and females may be significantly different. We believe that better understanding gender differences in diseases in general, and in asthma in particular, is of major importance in order to provide personalized education and management across the life course.

## Data Availability

All data generated or analysed during METAGEM - PROXIMA study are included in this published article.

## Abbreviations

ACQ: Asthma Control Questionnaire
B-IPQ: Brief Illness Perception Questionnaire
EQ-5D-3 L: EuroQoL five-dimensional three-level questionnaire
GINA: Global Initiative for Asthma
ICS: inhaled corticosteroids
IQR: interquartile range
LABA: long-acting beta2-agonists
OCS: oral corticosteroids
OR: odds ratio
PROs: patient reported outcomes
QoL: quality of life
SAA: severe allergic asthma
SD: standard deviation
